# A semi-supervised learning approach to predict synthetic genetic interactions by combining functional and topological properties of functional gene network

**DOI:** 10.1186/1471-2105-11-343

**Published:** 2010-06-24

**Authors:** Zhu-Hong You, Zheng Yin, Kyungsook Han, De-Shuang Huang, Xiaobo Zhou

**Affiliations:** 1 Intelligent Computing Lab, Institute of Intelligent Machine, Chinese Academy of Science, P.O. Box 1130, Hefei, Anhui 230031, China; 2 Department of Automation, University of Science and Technology of China, Hefei, Anhui 230027, China; 3 The Methodist Hospital Research Institute, Weil Medical College, Cornell University, Houston, TX 77030, USA; 4 School of Computer Science and Engineering, Inha University, Incheon, South Korea

## Abstract

**Background:**

Genetic interaction profiles are highly informative and helpful for understanding the functional linkages between genes, and therefore have been extensively exploited for annotating gene functions and dissecting specific pathway structures. However, our understanding is rather limited to the relationship between double concurrent perturbation and various higher level phenotypic changes, e.g. those in cells, tissues or organs. Modifier screens, such as synthetic genetic arrays (SGA) can help us to understand the phenotype caused by combined gene mutations. Unfortunately, exhaustive tests on all possible combined mutations in any genome are vulnerable to combinatorial explosion and are infeasible either technically or financially. Therefore, an accurate computational approach to predict genetic interaction is highly desirable, and such methods have the potential of alleviating the bottleneck on experiment design.

**Results:**

In this work, we introduce a computational systems biology approach for the accurate prediction of pairwise synthetic genetic interactions (SGI). First, a high-coverage and high-precision functional gene network (FGN) is constructed by integrating protein-protein interaction (PPI), protein complex and gene expression data; then, a graph-based semi-supervised learning (SSL) classifier is utilized to identify SGI, where the topological properties of protein pairs in weighted FGN is used as input features of the classifier. We compare the proposed SSL method with the state-of-the-art supervised classifier, the support vector machines (SVM), on a benchmark dataset in *S. cerevisiae *to validate our method's ability to distinguish synthetic genetic interactions from non-interaction gene pairs. Experimental results show that the proposed method can accurately predict genetic interactions in *S. cerevisiae *(with a sensitivity of 92% and specificity of 91%). Noticeably, the SSL method is more efficient than SVM, especially for very small training sets and large test sets.

**Conclusions:**

We developed a graph-based SSL classifier for predicting the SGI. The classifier employs topological properties of weighted FGN as input features and simultaneously employs information induced from labelled and unlabelled data. Our analysis indicates that the topological properties of weighted FGN can be employed to accurately predict SGI. Also, the graph-based SSL method outperforms the traditional standard supervised approach, especially when used with small training sets. The proposed method can alleviate experimental burden of exhaustive test and provide a useful guide for the biologist in narrowing down the candidate gene pairs with SGI. The data and source code implementing the method are available from the website: http://home.ustc.edu.cn/~yzh33108/GeneticInterPred.htm

## Background

Genetic interaction analysis, in which two mutations have a combined effect not exhibited by either mutation alone, can reveal functional relationship between genes and pathways, and thus have been used extensively to shed light on pathway organization in model organisms [1,2]. For example, proteins in the same pathway tend to share similar synthetic lethal partners [3]. Given a pair of genes, the number of common genetic interaction partners of these two genes can be used to calculate the probability that they have physical interaction or share a biological function. Therefore, identifying gene pairs which participate in synthetic genetic interaction (SGI) is very important for understanding cellular interaction and determining functional relationships between genes. Usually, SGI includes synthetic lethal (SL, where simultaneous mutation, usually deletion, on both genes causes lethality while mutation on either gene alone does not) and synthetic sick (SS, where simultaneous mutation of two genes causes growth retardation) interactions. However, so far little is known about how genes interact to produce more complicated phenotypes like the morphological variations.

Recently, modifier screening such as synthetic genetic arrays (SGA) has been applied to experimentally test the phenotype of all double concurrent perturbation to identify whether gene pairs have SGI [3]. Although high-throughput SGA technology has enabled systematic construction of double concurrent perturbation in many organisms, it remains difficult and expensive to experimentally map out pairwise genetic interactions for genome-wide analysis in any single organism. For example, the genome of *S. cerevisiae *includes about 6,275 genes. About 18 million double mutants need to be tested if the analysis is carried out based on their combinatorial nature. This number will expand to about 200 million for the simple metazoan *C. elegans *(with ~20,000 genes), posing insurmountable technical and financial obstacles.

Therefore, many computational methods for predicting SGI have been proposed in previous works in order to alleviate the experimental bottleneck [4,5]. A promising solution is to predict the SGI by integrating various types of available proteomics and genomics data. Candidate gene pairs with SGI are computationally predicted and validated experimentally. In [4], SS or SL gene pairs in *S.cerevisiae *are successfully predicted, with 80% of the interactions being discovered by testing 20% of all possible combinations of gene pairs. Various supervised algorithms, such as artificial neural network, SVM and decision tree, have been developed to tackle the synthetic genetic interaction prediction problem [4,6]. In spite of being able to handle large input spaces and deal with noisy samples in an efficient and robust way, a main difficulty facing all supervised methods is that they predict the SGI only from labelled samples and the learning process heavily relies on the quality of the training dataset [7]. For example, in [4] about 519,647 experimentally tested gene pairs of *S. cerevisiae *are adopted as training dataset, which is impossible in most cases.

Usually, obtaining labelled samples is much more difficult than getting unlabelled samples. When the size of available training set is small, traditional approaches based on supervised learning may fail. Worse still, experiment-supported genetic interactions gene pairs are far more less in metazoans than in *S. cerevisiae*, thus it is more difficult in metazoans to generate genome-wide predictions by using supervised algorithms. Therefore, it is desirable to develop a predictive learning algorithm using both labelled and unlabelled samples. In this context, it becomes natural that semi-supervised classifiers are employed. SSL classifier uses available label information as well as the wealth of unlabelled data as the input vector. We propose a graph-based SSL method, previously presented in [8], in the context of SGI prediction. One advantage of SSL is the compatibility to small training sets, thus it could have great potentials in organisms, especially metazoans with less experiment-supported genetic interaction gene pairs. We concentrate on graph-based method due to their solid mathematical background, as well as the close relationship with kernel methods and model visualization.

In recent years, it has been a growing and hot topic to combine information from diverse genomic or proteomic evidence in order to arrive at accurate and holistic network [9-13]. The heterogeneous data sources, in one way or the other, carry interaction information reflecting different aspects of gene associations and their function relationships. Therefore, one of the major challenges is to integrate these data sources and obtain a system level view on functional relationships between genes [14]. The successful applications have proved that an integration of heterogeneous types of high-throughput biological data can improve the accuracy of the groupings compared with any single dataset alone [10,15-19]. However, despite the success of integrated networks in other area [10], most previous works on synthetic genetic interaction prediction mainly consider PPI or gene expression data alone [20-22].

In this study, we integrate PPI, protein complex and gene expression data simultaneously to utilize more information for more accuracy of genetic interaction prediction taking the following observations into consideration. PPI data is believed to contain valuable insight for the inner working of cells. Therefore, it may provide useful clues for the function of individual protein or signalling pathways [23,24]. Although it is unclear which proteins are in physical contact, protein complexes include groups of proteins perform a certain cellular task together and contain rich information about functional relationships among the involved proteins. The high-throughput gene expression profiles are becoming essential resources for systems-level understanding of genetic interaction [25-28]. Gene expression profiles measure the expression levels of certain genes in genome scale. Relative to randomly paired genes, functionally interacting genes are more likely to have similar expression patterns and phenotypes [5,29,30]. It is assumed that genes with similar expression profiles are involved in the control of the same transcriptional factors and thus they are functionally associated [25,31].

Network analysis is a quantitative method originating from the social science; it studies the nodes' topology properties related to its connectivity and position in the network. It has become increasingly popular in diverse areas, especially in molecular biology and computational biology [9,32]. Network analysis is a powerful tool for studying the relationships between two nodes in a network. It has been proved in recent work that genetic interactions are more likely to be found among proteins that are highly connected and highly central in protein interaction network [33]. This finding demonstrates the correlations between topological properties of PPI network and SGI between proteins. In this study, we study the extent to which pairwise SGI can be predicted from the topological properties of the corresponding proteins in a FGN.

In previous works, they only consider the topological properties of the binary protein interaction network while ignore the underlying functional relationships which can be reflected by the gene expression profile [4,20]. A major limitation of these methods stems from the fact that the weight of ties is not taken into account. For FGN, the weights often reflect the function similarity performed by the ties. Exploring the information that weights hold allows us to further our understanding of networks [34,35]. In this paper, we also present a straightforward generalization of a number of weighed network properties which originally defined on the unweighted networks. Concretely, the weighted network properties are defined by combining weighted and topological observables that enable us to characterize the complex statistical properties and heterogeneity of the actual weight of edges and nodes. This information allows us to investigate the correlations among weighted quantities and the underlying topological structure of the network. The topological properties of the FGN are examined with the aim of discovering the relationship between the network properties of gene pairs and the existence of a SGI relationship.

## Results

### General approach

**Figure 1:**
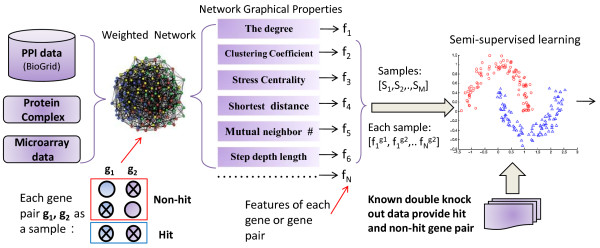
**Schematic diagram for predicting the synthetic genetic interaction**. The simplest case of a synthetic genetic interaction and non-interaction involving two genes is shown (See low left corner of Fig. 1). The green and purple nodes denote two different genes. The node with cross inside means it is knocked out and vice versa. Here, two single deletions would not result in cell mortality but would result in a "synthetic" lethality phenotype. Hit gene pair means mutations in both of these two genes would result in a "synthetic" lethality phenotype.

The aim of the proposed approach is to predict genetic interactions in *Saccharomyces cerevisiae *using topological properties of two proteins in a weighted functional gene network. The first input feature vector for the algorithm is a set of network properties corresponding to pairwise genes. The second input is a set of synthetic genetic interaction and non-interaction pairs found from previous large scale mutant screens. The output of this approach is scores corresponding to the propensity of a particular gene pair to be synthetic genetic interaction. The overall workflow is illustrated in Figure 1.

We can see from Fig. 1 that PPI data, protein complex data, and gene expression profiles are integrated to build a high coverage and high precision weighted FGN. More specifically, PPI and protein complex data are used to determine the topology of the network. Then a clustering analysis method is utilized to identify functionally related groups from the gene expression profile and the weights of the interaction are calculated based on the gene expression profile and clustering centroids, i.e. the weight of a PPI network derives from a metric considering the distance of expression of individual gene and the centroids of its cluster, as well as the distance between the two cluster centroids themselves. The weights are assigned as the confidence scores which represents their functional coupling. Considering weights of interactions instead of binary linkage information allows more accurate modelling and will have better classification performance [15,17].

And then, a set of topological properties are extracted from the FGN. These network properties and the experimentally obtained gene pairs which have been confirmed to have or do not have the synthetic genetic interaction are considered as an input vector of a SSL classifier to predict other unknown interacting gene pairs. Concretely, we use a SSL classifier to model correlations between network properties and the existence of a SGI. The output labels of the SSL classifier are soft labels *y*_*i *_∈ [0, 1], which measure if the two corresponding genes participate in a SGI. The details of above procedure are described in the method section.

### Cross validation

Performance comparisons are based on the following Cross Validation (CV) procedures. CV is a way of choosing proper benchmarking samples to assess the accuracy and validity of a statistical model. Specifically, we randomly select 1,500 known SGI pairs and 1,500 non-SGI pairs from the dataset provided by Tong *et al *[3]. Thus, the sampled dataset contain an equal number of SGI and non-SGI gene pairs. In *n *- *fold *CV, we randomly divide the known SGI pairs into *n *subsets of approximately equal size. Equal number of non-SGI pairs corresponding to above *n *divided subsets are randomly selected and assigned to the *n *subsets. Then *n *- 1 such subsets are combined for training the classifier, which is subsequently tested on all other SGI and non-SGI pairs from the withheld subset. This procedure is repeated *n *times with each subset playing the role of the test subset once.

We use the standard Receiver Operating Curve (ROC) to assess performance overall. We compute the sensitivity (or true-positive rate, defined here as the fraction of SGI gene pairs correctly predicted) and false-positive (defined here as the fraction of non-SGI gene pairs incorrectly predicted to be SGI) by decreasing stringency levels of the classifier (outputs soft labels). By using alternative score thresholds, this approach can be tuned to predict a subset of SGI with higher confidence at a small cost of sensitivity.

### Experiment results

SVM has emerged as one of the most popular supervised approaches with a wide range of applications. In particular, the previous studies have demonstrated that SVM has better learning performance and accuracy than other supervised algorithms, such as Artificial Neural Network and Decision Trees [36]. Therefore, in this study we implemented our graph-based SSL algorithm and compared it with the SVM in distinguishing SGI versus non-SGI gene pairs on the same benchmark dataset. We test the capability of our method using different levels of sparsity of training set. In the experiment, 80% (5-fold CV), 50% (2-fold CV), and 20% of the known SGI and non-SGI gene pairs are randomly chosen for training the classifier respectively, which was subsequently tested on all other SGI and non-SGI gene pairs from the withheld group (This is repeated several times with each group playing the role of the test group at least one time). Since the gene pairs to be classified for cross-validation are randomly chosen, we repeated each experiment five times and computed the average of all the results.

**Figure 2:**
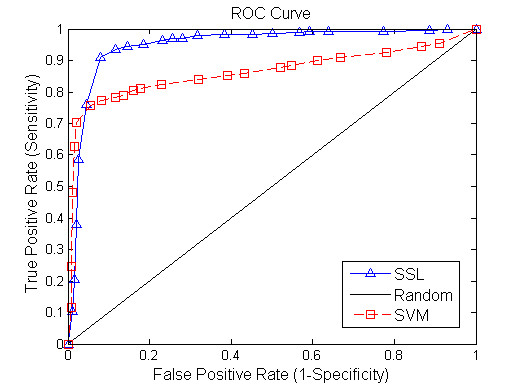
**Comparison of the ROC curves for Semi-supervised learning and SVM algorithm (unknown genetic interaction and non-interaction gene pairs: 20%)**. The horizontal axis is 1-Specificity and vertical axis is the corresponding Sensitivity. The diagonal line denotes random prediction.

**Figure 3:**
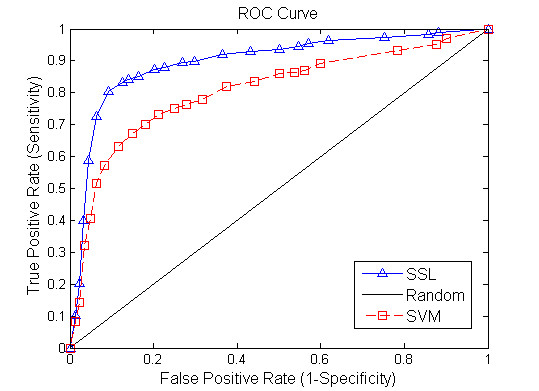
**Comparison of the ROC curves for Semi-supervised learning and SVM algorithm (unknown genetic interaction and non-interaction gene pairs: 50%)**. The horizontal axis is 1-Specificity and vertical axis is the corresponding Sensitivity. The diagonal line denotes random prediction.

**Figure 4:**
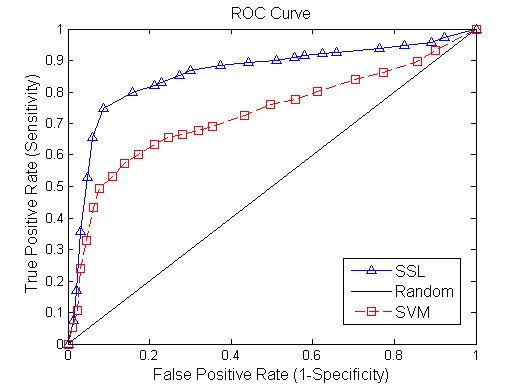
**Comparison of the ROC curves for Semi-supervised learning and SVM algorithm (unknown genetic interaction and non-interaction gene pairs: 80%)**. The horizontal axis is 1-Specificity and vertical axis is the corresponding Sensitivity. The diagonal line denotes random prediction.

Figure 2 shows a comparison result between SSL algorithm and SVM method when 20% of gene pairs are assumed to be unlabelled. Figures 3, 4 demonstrate the performance of the two tested algorithms when 50% and 80% of gene pairs are assumed to be unlabelled respectively. The proposed SSL algorithm outperforms SVM in almost all the range of threshold. In particular, we can see from Figure 2 that when 20% of nodes are unlabelled, SVM has a slightly better performance in the first part of the ROC curve while SSL achieves better results in other part. Conversely, when 80% of nodes are unlabelled, SSL shows much higher accuracy than SVM (see Figure 4). In summary, the accuracy of SSL appears higher than that of SVM classifier. Further, when labelled nodes in training dataset are very small, the performance of SSL is significantly better than that of SVM. SSL method can reach a true positive rate of 92% against a false positive rate of 9% accuracy at a maximum in our experiment. However, Wong *et al *[4] reported that they predicted SSL gene pairs in *S. cerevisiae *with a success rate such that 80% of the interactions are discovered by testing <20% of the pairs. Our algorithm has higher accuracy than their method. Moreover, our approach only depends on protein interaction data and gene expression data, and does not require other data source like genomic sequence data. Our results clearly demonstrate that the FGN integrating of proteome and genomic data can be used to predict the SGI. We exhibit that the topological properties of FGN for pairwise genes serve as compelling and relatively robust determinants for the existence of synthetic genetic interaction between genes.

**Figure 5:**
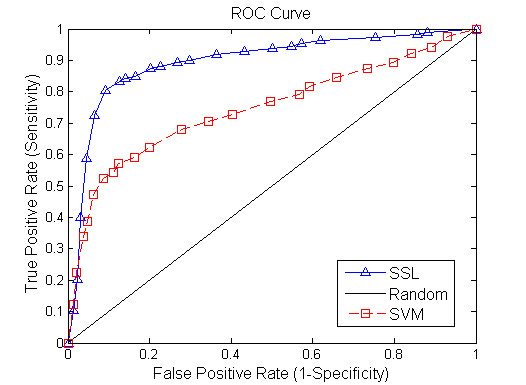
**Semi-supervised Learning performance comparison between the weighted functional gene network and the binary network (unknown genetic interaction and non-interaction gene pairs: 50%)**. The horizontal axis is 1-Specificity and vertical axis is the corresponding Sensitivity. The diagonal line denotes random prediction.

As a supplementary result we also compare the performance of proposed method on the same training dataset between the weighted network and binary network. The binary network is constructed by combining PPI and protein complex data. From Figure 5, we can see that the weighted network has higher performance than that of binary network in almost all the range of threshold. We believe this is because for binary network the weights of interactions are not taken into account and the information that weights hold is not employed.

## Discussion

**Figure 6:**
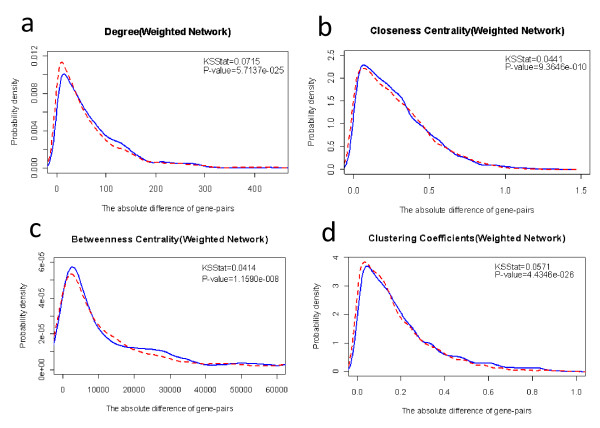
**The distribution of the absolute difference of (a) degree centrality, (b) closeness centrality, (c) betweenness centrality, (d) clustering coefficient across a pair of genes in case of the synthetic genetic interaction pairs (blue line) and Non-Synthetic genetic interaction pairs (red dashed line) in the weighted network (functional gene network)**. Numbers in each plot indicate the D-statistic associated with the Kolmogorov-Smirnov test for the difference between the two distributions and the corresponding P-value.

In order to assess the suitability of using certain network properties to classify SGI gene pairs and non-SGI pairs, we draw the distributions of probability density for these properties across SGI pairs and non-SGI pairs, respectively. To make it simple, we just give detailed description of four network properties, such as centrality degree, betweenness centrality, closeness centrality and clustering coefficient. For each property, we plot the distribution of the average value over pairwise genes and the absolute difference across the two genes. For most properties here, the difference in distribution of probability density across SGI and non-SGI pairs is statistically significant (see Additional file 1 Figure S1-S16). The distributions of the average and difference value of each property across two proteins in case of SGI pairs (blue lines) and non-SGI pairs (red lines) are displayed in figure 6.

We used the Kolmogorov-Smirnov (K-S) test to compare the two distributions. The null hypothesis is that the two distributions are from the same continuous distribution. The alternative hypothesis is that they are from different continuous distributions. The major contribution of K-S test is that no distribution assumption is needed for the data. As shown in Table 1 that all the P-values of the KS-test are less than 0.05. From Additional file 1 Figure S17-S32, we can see that the empirical distributions of cumulative function across SGI and non-SGI pairs are also different. According to the result, the difference between SGI and non-SGI samples is significant enough. Also when viewed as part of a FGN, comparing with non-SGI pairs, SGI pairs tends to have higher average degree, higher average closeness centrality. We also compared the KS-test performance in weighted FGN and binary network. We can see from Table 1 that the P-values of all network properties in weighted network are much less than those in binary network.

**Table 1:** The statistics of network properties for SGI vs. non-SGI gene pairs

Gene pair characteristic		KSStat		P-value	
		
		Binary Network	Weighted Network	Binary Network	Weighted Network
Average	Degree	0.0364	0.0261	0.9	0.0011
	
	Closeness	0.0212	0.0385	0.0108	1.48E-07
	
	Betweenness	0.0319	0.0529	1.55E-05	7.05E-14
	
	Clustering Coefficient	0.0679	0.0691	1.41E-23	2.365E-24

Absolute Difference	Degree	0.0587	0.0715	1.00E-17	5.71E-25
	
	Closeness	0.0587	0.0441	1.00E-17	9.365E-10
	
	Betweenness	0.0313	0.0414	2.35E-05	1.56E-08
	
	Clustering Coefficient	0.0615	0.0571	1.97E-19	4.435E-26

## Conclusions

In conclusion, a SSL prediction approach was proposed in this paper to predict SGI by combining functional and topological properties of FGN. Using a clustering-based data integration method, large-scale protein interaction data, protein complex data and multiple time-course gene expression datasets were combined in order to build FGN in yeast. Greater coverage and higher accuracy were achieved in comparison with previous high-throughput studies of PPI networks in yeast. Then, we show that topological properties of protein pairs in a FGN can be served as compelling and relatively robust determinants for the existence of synthetic genetic interaction between them. Finally, a graph-based SSL is utilized as a classifier to model correlations between FGN properties and the existence of a synthetic genetic interaction.

Our results clearly demonstrate that the proposed algorithm can achieve better performance comparing with previous methods. Our framework of feature representation is a general form, and it is straightforward to add other topological properties that are relevant to this problem. It is also possible to add other types of biological evidences. For example, information about the function of proteins can be encoded in our framework as well. We hope to extend this work and improve feature representation in future so that we can detect other types of interaction groups.

## Methods

### Biological datasets

There are four different types of data sets used in the study. 1) Golden standard dataset of known genetic interactions (True positives, TPs) and non-interacting protein pairs (True negatives, TNs). 2) Experimental protein-protein interaction data. 3) Experimental protein complex data. 4) Time-lapse gene expression profiles.

#### Golden standard genetic interaction dataset

Using the Synthetic Genetic Array (SGA) technology, Tong et al. screened 132 query strains (carrying mutations in genes with diverse functions in cell polarity, cell wall biosynthesis, chromosome segregation and DNA synthesis and repair) against the complete library of ~4700 viable haploid deletion strains, and ~650,000 gene pairs were experimentally tested and identified a total of ~4,000 synthetic lethal synthetic sick interactions, at 0.65% frequency [3]. We used this dataset as golden standard dataset to investigate synthetic genetic interaction in *S. cerevisiae*.

#### Protein-protein interaction dataset

To computer network properties associated with protein-protein interaction in *S. Cerevisiae*, we download protein interaction data from the BioGrid database [37]. This network contains 12,990 unique interactions among 4,478 proteins.

#### Protein complexes dataset

For protein complex, we assigned binary interactions between any two proteins participating in a complex. Thus in general, if there are *n *proteins in a protein complex, we add *n*(*n *- 1)/2 binary interactions. We get the protein complex data from [38,39]. Altogether about 49,000 interactions are added to the protein interaction network.

#### Microarray gene expression data

Four sets of time course data from the DNA microarray of *S. cerevisiae *are used in this study. These datasets have also been used to study the genetic interactions in previous work [40]. The first set contains 17 time points during the mitotic cell cycle [41]. The second set contains 6 time points during heat shock and the third set contains 9 time points during sporulation [31], and the fourth set contains 32 time points during cell cycle [42]. Altogether 64 experimental conditions for all the genes in *S. cerevisiae *related to cell cycle are used. For the missing values in each experiment, we substituted its gene expression ratio to the reference state with the average ratio of all the genes under that specific experimental condition.

### Construction of functional gene networks

Linkages of the FGN carry confidence scores to represent the functional coupling between two biological entities they represent. In this section, we calculated the confidence score of each linkage following the previous works [25,26].

For the gene expression data, the clustering analysis is carried out to identify functionally related groups of genes. We denote a gene expression data set as *X *= {*x*_1_, *x*_2_,...,*x*_*M*_}, where *x*_*i *_= {*x*_*i*1_, *x*_*i*2_,...,*x*_*iN*_} is a *N *dimensional vector representing gene *i *with *N *conditions. We use the clustering algorithm to group the *M *genes into *S*,(*S *≤ *M *-1) different clusters *C*_1_, *C*_2_,...,*C*_*s*_.

As proposed in [43], the Pearson Correlation Coefficient (PCC) is employed as a measure of similarity to cluster genes with similar or different expression patterns, which means genes with co-expressed pattern are assigned to same cluster and vice versa. A positive PCC value means that two genes are co-expressed, while negative value denotes that they are the opposite expressed gene pairs. Let us consider genes *x*_*i *_and *x*_*j *_and the PCC can be calculated as(1)

where *x*_*ik*_and *x*_*jk*_are the expression values of the *kth *condition of the *ith *and *jth *genes respectively.  and  are the mean values of the *ith *and *jth *genes respectively. PCC is always in the range of (-1, 1).

At first, all genes of the gene expression profiles are considered as a single cluster and the cluster is partitioned into two disjoint clusters. Partitioning is done in such way that *x*_*i *_and *x*_*j *_which have the most negative value of PCC will be assigned into two different clusters. Genes having larger PCC value with *x*_*i *_compared with *x*_*j *_are assigned in the cluster that contains *x*_*i*_. Otherwise, they are placed in the cluster that contains *x*_*j*_. In the next iteration, a cluster having a gene pair (*x*_*i*_, *x*_*j*_) with the most negative PCC value will be selected and the above partitioning procession is repeated until there is no negative PCC value present between any pair of genes inside any cluster. This kind of cluster method ensures that all pairs of genes in any cluster are only positively correlated. It has been proven that this method is able to obtain clusters with higher biological significance than that obtained by some other algorithms such as Fuzzy K-means, GK and PAM clustering methods [43].

Based on the above obtained gene expression profile which has been partitioned into a couple of clusters, we calculate the weighted confidence scores of the interactions between two proteins as below:(2)

where *x*_*i *_and *x*_*j *_represent genes *i *and *j *with *N *conditions respectively.  and  denote the centroids of the clusters in which genes *x*_*i *_and *x*_*j *_located respectively. ||·||^2 ^denotes the Euclidean distance. In equation (2), the constant *L*_1 _is a tradeoff parameter used to tune the ratio of the first and second term in the weight function. According to [44], we choose *L*_1 _= 0.3 because we assume that the distance between centroids of two cluster more significant comparing with the distance of each gene from its centroid. The outcome of the integration method is a weighted undirected graph, i.e. functional gene network.

### The properties of functional gene network for predicting SGI

For using as input feature vector of the SSL classifier, we compute the following topological properties of FGN for each protein or protein pair. Here we report a total of 18 features representing 10 network properties. These network properties reflect the local connectivity and global position of the nodes in the network and are assumed to be correlated to its functional properties. Table 2 lists the 10 types of topological properties used in this paper. The details can be seen as below.

**Table 2:** Features for representing synthetic interaction

	Gene pair characteristic	Reference	Graph Type
1	**Centrality degree**	Barrat et al. (2004)	Weight

2	**Clustering coefficient**	Barrat et al. (2004).	Weight

3	**Betweenness centrality**	Brandes. (2001)	Weight

4	**Closeness centrality**	Newman. (2001)	Weight

5	**Eigenvector centrality**	Csardi G. (1965)	Weight

6	**Stress centrality**	Freeman LC. (1977)	Binary

7	**Information centrality**	Stephenson K. (1989)	Binary

8	**Shortest path length**	Newman. (2001)	Weight

9	**Flow between centrality**	Newman. (2001)	Binary

10	**Mutual neighbor**	Newman. (2001)	Binary

#### (1) Centrality degree

A network can be expressed by its adjacency matrix *a*_*ij*_, whose elements take the value 1 if an edge connects the node *v*_*i *_to the node *v*_*j *_and 0 otherwise. In an unweighted graph, the degree of node *v*_*x *_is equivalent to the number of neighbors of node *v*_*x *_, which can be denoted as(3)

However, the weighted degree of node *v*_*i *_is the sum of the weights of the edges between *v*_*i *_and its neighbors [45].(4)

where *ω *is the weight between two nodes, in which *ω*_*ij *_is greater than 0 if node *v*_*i *_is tied to node *v*_*j *_, and the value is the weight of the tie, which represents the strength of the relation between the two nodes.

#### (2) Clustering coefficient

The clustering coefficient of a node in a network quantifies how close the node and its neighbors are to being a clique. Let *C*_*cl*_(*i*) denote the clustering coefficient of node *v*_*i *_, and it is given by the proportion of links between the nodes within its neighbourhoods divided by the number of links that could possibly exist between them. For an unweighted graph, the clustering coefficient can be defined as:(5)

where *e*_*i *_is the number of the links between the neighbourhoods of node *i *and *k*_*i *_is the number of the neighbourhoods of node *v*_*i *_. For a weighted graph, the definition of the clustering coefficient is defined as [45](6)

#### (3) Weighted Shortest Path

Both the closeness centrality and betweenness centrality rely on the calculation of shortest path in a network. Therefore, a first step towards extending these measures to weighted networks is to generalize how shortest path is defined in weighted networks.

In weighted networks, the shortest path is a path between two nodes with the minimal sum of the weights of its constituent edges. Since all edges have the same weight in unweighted networks, the shortest path between two nodes is through the smallest number of intermediary nodes. However, a complication arises when the ties in a network do not have the same weight attached to them. There have been several attempts to calculate shortest distances in weighted networks in previous work [46,47]. In our work, we applied Dijkstra's algorithm to the weighted biological network by inverting the positive weights in the network [47]. Thus, high values represent weak ties, whereas low values represent strong ties.

#### (4) Betweenness centrality

Betweenness is a centrality measure of a node within a networks. Nodes that occur on many shortest paths between other nodes have higher betweenness than those that do not. For an unweighted network, to calculate the betweenness  of node *v*_*i*_, we firstly count the number of shortest paths between two nodes passing the node *v*_*i *_. Let *b*_*i *_be the ratio of this number to the total number of shortest paths existing between these two nodes. Then the betweenness of node *v*_*i *_is the sum of *b*_*i *_over all pairs of nodes in the network. We normalize it to lie between 0 and 1 by dividing above value by the total number of pairs in the network. The betweenness for node *v*_*i *_is as follow(7)

where *g*_*jk *_is the number of shortest geodesic paths from node *v*_*j *_to *v*_*k*_. *g*_*jk*_(*i*) is the number of shortest geodesic paths from *v*_*j *_to *v*_*k *_which pass through the node *v*_*i*_.

In the case of weighted network, we assume that the flow in the network occurs over the paths that Dijkstra's algorithm identifies and use this algorithm to find the nodes that funnel the flow in the network. Then the weighted betweenness centrality is extended by counting the number of paths found by Dijkstra's algorithm on a weighted network instead of the number found on a binary network [48].

#### (5) Closeness centrality

Closeness is a centrality measure of a node within a network. Nodes which tend to have short geodesic distances to all other nodes within the network have higher closeness. In unweighted network, closeness centrality is defined as the inverse of the average distance from one node to all other nodes. For a weighted network, this definition changes slightly. Within the adjacency matrix, for any two nodes, *v*_*i *_and *v*_*j *_, if *d*_*ij *_is the shortest distance from *v*_*i *_to *v*_*j*_, then the closeness centrality of node *v*_*j *_is defined as [49](8)

where *n *is the total number of nodes in the network.

#### (6) Eigenvector centrality

Eigenvector centrality is a measure of the importance of a node in a network. It assigns relative scores to all nodes in the network based on the principle that connections to high-scoring nodes contribute more to the score of the node in question than equal connections to low-scoring nodes.

Let *x*_*i *_denotes the score of the *ith *node. Let *A*_*ij *_be the adjacency matrix of the network. In weighted network, the entries of *A *are real numbers representing connection strengths. For the *ith *node, let the eigenvector centrality score be proportional to the sum of the scores of all nodes which are connected to it. It can be formulated as [49]:(9)

where *λ *is a constant. Defining the vector of centralities *x *={*x*_1_,*x*_2_,...,*x*_*n*_}, we can rewrite this equation in matrix form as(10)

Hence we see that *x *is an eigenvector of the adjacency matrix with eigenvalue *λ*. In our work, we used the free software package named igraph to calculate the eigenvector centrality of weighted network [50].

In addition to above six weighted network properties, we also calculated several other binary network properties, such as stress centrality [51], information centrality [52], flow betweenness centrality [53], the number of mutual neighbors between proteins *v*_*i *_and *v*_*j*_. All of the above ten network properties can reflect the local network structure around the node or the global network topology.

### Graph-based semi-supervised classifier

The SSL is halfway between supervised and unsupervised learning, which is very active and has recently attracted a considerable amount of research [7,54]. In essence, there are three different kinds of SSL algorithms being applied, i.e., Generative models, Low density separation algorithms, and Graph-based methods. In our study, we use graph-based SSL method because of its solid mathematical background, their relationship with kernel methods, visualization, and good results in many areas, such as computational biology [32], web page classification [54], or hyperspectral image classification [7]. We here present the whole formulation of the graph-based SSL algorithm.

Consider the whole dataset being represented by *χ *= (*χ*_*l*_, *χ*_*n*_) of labelled inputs *χ*_*l *_= {*x*_1_, *x*_2_,...,*x*_*l*_} and unlabelled inputs *χ*_*n *_= {*x*_*l*+1_, *x*_*l*+2_,...,*x*_*n*_} along with a small portion of corresponding labels {*y*_1_, *y*_2_,...,*y*_*l*_}. Consider a connected weighted graph *G *= (*V*, *E*) with vertex *V *corresponding to above *n *data points, with nodes *L *= {1, 2,...,*l*} corresponding to the labelled points with labels *y*_1_, *y*_2_,...,*y*_*l *_and *U *= {*l *+ 1, *l *+2,...,*n*} corresponding to unlabelled points. For SSL, the objective is to infer the labels {*y*_*l*+1_, *y*_*l*+2_,...,*y*_*n*_} of the unlabelled data {*x*_*l*+1_, *x*_*l*+2_,...,*x*_*n*_}, typically *l *≪ *n*.

Firstly, the *n *× *n *symmetric weight matrix *W *on the edges of the graph can be(11)

where *x*_*i *_and *x*_*j *_denote the different points in the graph *G*. The constant σ is a length scale hyperparameter. Therefore nearby points in Euclidean spaces are assigned large edge weight, and vice versa.

Then let *F *denotes a series of *n *× *l *matrices with non-negative elements. A matrix  corresponds to one certain classification on *χ *= (*χ*_*l*_, *χ*_*n*_) by assigning each point *x*_*i *_a label *y*_*i *_= *argmax x*_*j*≤*l*_.*F*_*ij*_. We define an *n *× *l *matrix *Y *∈*F *with *Y*_*ij *_= 1 if *x*_*i *_is labelled as *y*_*i *_= *j *and *Y*_*ij *_= 0 otherwise.

Secondly, we build the matrix *S *= *D*^-1 2^*WD*^-1 2 ^where *D *is a diagonal matrix with the (*i*, *i*) -elements equal to the sum of the *ith *row of *W*. Then take the iteration *F*(*t *+ 1) = *αSF*(*t*) + (1 - *α*)*Y *until the similarity matrix *F *converges, where *α *is a predefined constant which ranges from 0 to 1.

Thirdly, let F* represent the limit of the sequence {*F*(*t*)}. Label each point *x*_*i *_as a label *y*_*i *_= *argmax x*_*j*≤*C*_.F*_ij_. Because 0 <*α *< 1 and the eigenvalues of *S *ranges from -1 to 1.(12)

Then the classification matrix can be calculated as: F* = (1 - α*S*)^-1^*Y*. As in [8], F* can be obtained without iteration. After the above steps, the labels of unlabelled data {*x*_*l*+1_, *x*_*l*+2_,...,*x*_*n*_} will be assigned.

### Support vector machines classifier

SVM algorithm has been proposed by Vapnik as an effective and increasingly popular learning approach for solving two-class pattern recognition problems [55]. SVM as a typical supervised machine learning method is attractive because it is not only well founded theoretically, but also superior in practical applications. Intuitively, SVM classifier is based on the structure risk minimization principle for which error bound analysis has been theoretically motivated. The method is defined over a vector space where the problem is to find a decision surface that "best" separates the data points in two classes by finding a maximal margin. SVM has been widely applied to a number of pattern recognition areas like text categorization [56], object recognition [57], etc. In most of these cases, the performance of SVM is significantly better than that of other supervised machine learning methods, including Neural Network and Decision Tree classifier [17]. The SVM has a number of advanced properties, including the ability to handle large feature space, effective avoidance of overfitting, and information condensing for the given data set, etc. A brief introduction about SVM is given in the Additional file 1.

Here, we describe the use of the LibSVM provided by Chih-Chung Chang. LibSVM is an integrated software for support vector classification [58]. It is much easy to construct a SVM classifier. We only need to choose a kernel function and regularization parameter to train the SVM. In this study, we adopt the radial basis function (RBF) as the kernel function whose parameters were optimized by taking a n-fold cross-validation on the training set [55]. Specifically, the grid search was used to find optimal kernel parameters such as *C, Gamma*, which tries values of each parameter across the specified search range using geometric steps. Although grid search method is computationally expensive, it is computationally feasible in our cases.

## Authors' contributions

ZHY & XBZ & DSH conceived the original idea, wrote the main body of the manuscript and implemented the experiments. ZY & KH attended the discussion of the work and revised the manuscript. All authors have read and approved the final version of this manuscript.

## Supplementary Material

Additional file 1 **This file consists of three parts of supplementary materials**. The first part contains a more detailed description of SVM and the parameters settings used in this study. The second part contains figures which show the probability density distribution of different network properties across synthetic genetic interactions and non-interaction pairs. The third part contains figures that show the empirical cumulative distributions of different network properties across synthetic genetic interactions and non-interaction pairs. Click here for file
