# Supplementary material for: A semi-supervised learning approach to predict synthetic genetic interactions by combining functional and topological properties of functional gene network
Source: BMC Bioinformatics. 2010 Jun 24;11:343. doi: 10.1186/1471-2105-11-343 (PMC2909217; doi:10.1186/1471-2105-11-343)
Supplement: Additional file 1 — This file consists of three parts of supplementary materials. The first part contains a more detailed description of SVM and the parameters settings used in this study. The second part contains figures which show the probability density distribution of different network properties across synthetic genetic interactions and non-interaction pairs. The third part contains figures that show the empirical cumulative distributions of different network properties across synthetic genetic interactions and non-interaction pairs. [file 1471-2105-11-343-S1.DOC]

A semi-supervised learning approach to predict synthetic genetic interactions by combining functional and topological properties of functional gene network

### Zhu-Hong You^1, 2, 3^, Zheng Yin^3^, Kyungsook Han^4^, De-Shuang Huang^1§^ and Xiaobo Zhou ^3§^

^1^Intelligent Computing Lab, Institute of Intelligent Machine, Chinese Academy of Science, P.O. Box 1130, Hefei, Anhui 230031China

^2^Department of Automation, University of Science and Technology of China, Hefei, Anhui 230027, China

^3^The Methodist Hospital Research Institute, Weill Medical College, Cornell University, Houston, TX 77030, USA

^4^School of Computer Science and Engineering, Inha University, Incheon, South Korea

**Part One:** Brief descriptions of the SVM classifier

The SVM problem can be solved using quadratic programming techniques, using an optimization algorithm where the working set selection is based on steepest feasible descent. SVM has many advanced properties, including the ability to handle large feature space, effective avoidance of overfitting, etc. Specifically, the quadratic programming problem can be formulated as:


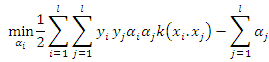


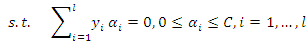


where
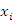
 denotes an input vector,
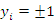
 corresponding to whether
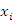
 belongs to the
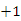
 class or
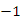
 class, e.g. synthetic genetic interaction class or non-interaction class in our case.
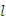
 presents the number of training sample.
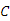
 is a regularization parameter that controls the trade off between margin and classification error.
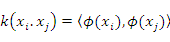
 represents the kernel function.
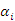
 is the solutions of the dual formulation. An unlabeled input vector
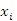
can be classified by the below discriminant function.


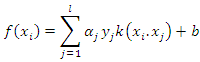


The input vector
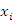
 is classified to the
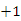
 or
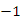
 class, e.g. synthetic genetic interaction class or non-interaction class in our case, if
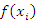
 is positive and vice versa.

parameters setting: Choosing a correct kernel is no free-lunch and the research is ongoing on optimizing the kernel design. The kernel functions can be linear or non-linear (Gaussian). The linear kernel function reduces to a linear equation on the original attributes in the training data. Based on our experience, linear kernel works well when there are many attributes (more that 100) in the training data, otherwise the Gaussian (RBF) kernel is used.

The Gaussian (RBF) kernel non-linearly maps samples into a higher dimensional space, unlike the linear kernel, can handle the case when the relation between class labels and attributes is non-linear. Actually, most of researchers suggest that in general RBF kernel is a reasonable first choice (REF: A Practical Guide to Support Vector Classification. Chih-Wei Hsu, Chih-Chung Chang, and Chih-Jen Lin ). Furthermore, the linear kernel is a special case of RBF (REF: Keerthi SS, Lin CJ: Asymptotic behaviors of support vector machines with Gaussian kernel. Neural Comput 2003, 15(7):1667-1689). In our case, the prior knowledge of our problem at hand guides us to choose Gaussian (FBF) kernel.

SVM with the Gaussian (RBF) kernel have been popular for practical use. Model selection in this class of SVM involves two hyperparameters: the penalty parameter C and the kernel width Gamma. If complete model selection using the Gaussian kernel has been conducted, there is no need to consider linear SVM. A grid search method can be used to try values of each parameter across the specified search range using geometric steps. Grid searches are computationally expensive because the model must be evaluated at many points within the grid for each parameter. For example, if a grid search is used with 10 search intervals and an RBF kernel function is used with two parameters (C and Gamma), then the model must be evaluated at 10*10 = 100 grid points. The grid search will find a region near the global optimum point. In our case, it is with an affordable computational complexity. Using the linear kernel it is also need to search for a parameter C. Actually the RBF is at least as good as linear. In current work, we think the SVM with RBF is enough for a baseline comparison.

**Part Two:** Figures show the probability density distribution of different network properties across synthetic genetic interactions and non-interaction gene pairs.

Supplementary Figure S1

The figure shows the probability density distribution of the average of degree centrality across a pair of genes in case of the synthetic genetic interaction pairs (blue line) and non-synthetic genetic interaction pairs (red dashed line) in the binary network (protein interaction network). Numbers in each plot indicate the D-statistic associated with the Kolmogorov-Smirnov test for the difference between the two distributions and the corresponding P-value.

Supplementary Figure S2

The figure shows the probability density distribution of the absolute difference of degree centrality across a pair of genes in case of the synthetic genetic interaction pairs (blue line) and non-synthetic genetic interaction pairs (red dashed line) in the binary network (protein interaction network). Numbers in each plot indicate the D-statistic associated with the Kolmogorov-Smirnov test for the difference between the two distributions and the corresponding P-value.

Supplementary Figure S3

The figure shows the probability density distribution of the average of closeness centrality across a pair of genes in case of the synthetic genetic interaction pairs (blue line) and non-synthetic genetic interaction pairs (red dashed line) in the binary network (protein interaction network). Numbers in each plot indicate the D-statistic associated with the Kolmogorov-Smirnov test for the difference between the two distributions and the corresponding P-value.

Supplementary Figure S4

The figure shows the probability density distribution of the absolute difference of closeness centrality across a pair of genes in case of the synthetic genetic interaction pairs (blue line) and non-synthetic genetic interaction pairs (red dashed line) in the binary network (protein interaction network). Numbers in each plot indicate the D-statistic associated with the Kolmogorov-Smirnov test for the difference between the two distributions and the corresponding P-value.

Supplementary Figure S5

The figure shows the probability density distribution of the average of betweeness centrality across a pair of genes in case of the synthetic genetic interaction pairs (blue line) and non-synthetic genetic interaction pairs (red dashed line) in the binary network (protein interaction network). Numbers in each plot indicate the D-statistic associated with the Kolmogorov-Smirnov test for the difference between the two distributions and the corresponding P-value.

Supplementary Figure S6

The figure shows the probability density distribution of the absolute difference of betweenness centrality across a pair of genes in case of the synthetic genetic interaction pairs (blue line) and non-synthetic genetic interaction pairs (red dashed line) in the binary network (protein interaction network). Numbers in each plot indicate the D-statistic associated with the Kolmogorov-Smirnov test for the difference between the two distributions and the corresponding P-value.

Supplementary Figure S7

The figure shows the probability density distribution of the average of clustering coefficient across a pair of genes in case of the synthetic genetic interaction pairs (blue line) and Non-synthetic genetic interaction pairs (red dashed line) in the binary network (protein interaction network). Numbers in each plot indicate the D-statistic associated with the Kolmogorov-Smirnov test for the difference between the two distributions and the corresponding P-value.

Supplementary Figure S8

The figure shows the probability density distribution of the absolute difference of clustering coefficient across a pair of genes in case of the synthetic genetic interaction pairs (blue line) and non-synthetic genetic interaction pairs (red dashed line) in the weighted network (functional gene network). Numbers in each plot indicate the D-statistic associated with the Kolmogorov-Smirnov test for the difference between the two distributions and the corresponding P-value.

Supplementary Figure S9

The figure shows the probability density distribution of the average of degree centrality across a pair of genes in case of the synthetic genetic interaction pairs (blue line) and non-synthetic genetic interaction pairs (red dashed line) in the weighted network (functional gene network). Numbers in each plot indicate the D-statistic associated with the Kolmogorov-Smirnov test for the difference between the two distributions and the corresponding P-value.

Supplementary Figure S10

The figure shows the probability density distribution of the absolute difference of degree centrality across a pair of genes in case of the synthetic genetic interaction pairs (blue line) and non-synthetic genetic interaction pairs (red dashed line) in the weighted network (functional gene network). Numbers in each plot indicate the D-statistic associated with the Kolmogorov-Smirnov test for the difference between the two distributions and the corresponding P-value.

Supplementary Figure S11

The figure shows the probability density distribution of the average of closeness centrality across a pair of genes in case of the synthetic genetic interaction pairs (blue line) and non-synthetic genetic interaction pairs (red dashed line) in the weighted network (functional gene network). Numbers in each plot indicate the D-statistic associated with the Kolmogorov-Smirnov test for the difference between the two distributions and the corresponding P-value.

Supplementary Figure S12

The figure shows the probability density distribution of the absolute difference of closeness centrality across a pair of genes in case of the synthetic genetic interaction pairs (blue line) and non-synthetic genetic interaction pairs (red dashed line) in the weighted network (functional gene network). Numbers in each plot indicate the D-statistic associated with the Kolmogorov-Smirnov test for the difference between the two distributions and the corresponding P-value.

Supplementary Figure S13

The figure shows the probability density distribution of the average of betweeness centrality across a pair of genes in case of the synthetic genetic interaction pairs (blue line) and non-synthetic genetic interaction pairs (red dashed line) in the weighted network (functional gene network). Numbers in each plot indicate the D-statistic associated with the Kolmogorov-Smirnov test for the difference between the two distributions and the corresponding P-value.

Supplementary Figure S14

The figure shows the probability density distribution of the absolute difference of betweenness centrality across a pair of genes in case of the synthetic genetic interaction pairs (blue line) and non-synthetic genetic interaction pairs (red dashed line) in the weighted network (functional gene network). Numbers in each plot indicate the D-statistic associated with the Kolmogorov-Smirnov test for the difference between the two distributions and the corresponding P-value.

Supplementary Figure S15

The figure shows the probability density distribution of the average of clustering coefficient across a pair of genes in case of the synthetic genetic interaction pairs (blue line) and non-synthetic genetic interaction pairs (red dashed line) in the weighted network (functional gene network). Numbers in each plot indicate the D-statistic associated with the Kolmogorov-Smirnov test for the difference between the two distributions and the corresponding P-value.

Supplementary Figure S16

The figure shows the probability density distribution of the absolute difference of clustering coefficient across a pair of genes in case of the synthetic genetic interaction pairs (blue line) and non-synthetic genetic interaction pairs (red dashed line) in the weighted network (functional gene network). Numbers in each plot indicate the D-statistic associated with the Kolmogorov-Smirnov test for the difference between the two distributions and the corresponding P-value.

**Figure S1.**

**Figure S2.**

**Figure S3.**

**Figure S4.**

**Figure S5.**

**Figure S6.**

**Figure S7.**

**Figure S8.**

**Figure S9.**

**Figure S10.**

**Figure S11.**

**Figure S12.**

**Figure S13.**

**Figure S14.**

**Figure S15.**

**Figure S16.**

**Part Three:** Figures show the empirical cumulative distributions of different network properties across synthetic genetic interactions and non-interaction gene pairs.

Supplementary Figure S17

The figure shows the empirical cumulative distribution of the average of degree centrality across a pair of genes in case of the synthetic genetic interaction pairs and non-synthetic genetic interaction pairs in the binary network.

Supplementary Figure S18

The figure shows the empirical cumulative distribution of the average of degree centrality across a pair of genes in case of the synthetic genetic interaction pairs and non-synthetic genetic interaction pairs in weighted functional gene network.

Supplementary Figure S19

The figure shows the empirical cumulative distribution of the absolute difference of degree centrality across a pair of genes in case of the synthetic genetic interaction pairs and non-synthetic genetic interaction pairs in the binary network.

Supplementary Figure S20

The figure shows the empirical cumulative distribution of the absolute difference of degree centrality across a pair of genes in case of the synthetic genetic interaction pairs and non-synthetic genetic interaction pairs in weighted functional gene network.

Supplementary Figure S21

The figure shows the empirical cumulative distribution of the average of closeness centrality across a pair of genes in case of the synthetic genetic interaction pairs and non-synthetic genetic interaction pairs in the binary network.

Supplementary Figure S22

The figure shows the empirical cumulative distribution of the average of closeness centrality across a pair of genes in case of the synthetic genetic interaction pairs and non-synthetic genetic interaction pairs in weighted functional gene network.

Supplementary Figure S23

The figure shows the empirical cumulative distribution of the absolute difference of closeness centrality across a pair of genes in case of the synthetic genetic interaction pairs and non-synthetic genetic interaction pairs in the binary network.

Supplementary Figure S24

The figure shows the empirical cumulative distribution of the absolute difference of closeness centrality across a pair of genes in case of the synthetic genetic interaction pairs and non-synthetic genetic interaction pairs in weighted functional gene network.

Supplementary Figure S25

The figure shows the empirical cumulative distribution of the average of betweenness centrality across a pair of genes in case of the synthetic genetic interaction pairs and non-synthetic genetic interaction pairs in the binary network.

Supplementary Figure S26

The figure shows the empirical cumulative distribution of the average of betweenness centrality across a pair of genes in case of the synthetic genetic interaction pairs and non-synthetic genetic interaction pairs in weighted functional gene network.

Supplementary Figure S27

The figure shows the empirical cumulative distribution of the absolute difference of betweenness centrality across a pair of genes in case of the synthetic genetic interaction pairs and non-synthetic genetic interaction pairs in the binary network.

Supplementary Figure S28

The figure shows the empirical cumulative distribution of the absolute difference of betweenness centrality across a pair of genes in case of the synthetic genetic interaction pairs and non-synthetic genetic interaction pairs in weighted functional gene network.

Supplementary Figure S29

The figure shows the empirical cumulative distribution of the average of clustering coefficient across a pair of genes in case of the synthetic genetic interaction pairs and non-synthetic genetic interaction pairs in the binary network.

Supplementary Figure S30

The figure shows the empirical cumulative distribution of the average of clustering coefficient across a pair of genes in case of the synthetic genetic interaction pairs and non-synthetic genetic interaction pairs in weighted functional gene network.

Supplementary Figure S31

The figure shows the empirical cumulative distribution of the absolute difference of clustering coefficient across a pair of genes in case of the synthetic genetic interaction pairs and non-synthetic genetic interaction pairs in the binary network.

Supplementary Figure S32

The figure shows the empirical cumulative distribution of the absolute difference of clustering coefficient across a pair of genes in case of the synthetic genetic interaction pairs and non-synthetic genetic interaction pairs in weighted functional gene network.

**Figure S17.**

**Figure S18.**

**Figure S19.**

**Figure S20.**

**Figure S21.**

**Figure S22.**

**Figure S23.**

**Figure S24.**

**Figure S25.**

**Figure S26.**

**Figure S27.**

**Figure S28.**

**Figure S29.**

**Figure S30.**

**Figure S31.**

**Figure S32.**
